# White Matter Alterations in Military Service Members With Remote Mild Traumatic Brain Injury

**DOI:** 10.1001/jamanetworkopen.2024.8121

**Published:** 2024-04-18

**Authors:** Sharon Kim, John Ollinger, Chihwa Song, Sorana Raiciulescu, Srija Seenivasan, Aaron Wolfgang, Hosung Kim, J. Kent Werner, Ping-Hong Yeh

**Affiliations:** 1 Program in Neuroscience, Uniformed Services University of Health Sciences, Bethesda, Maryland; 2 School of Medicine, Uniformed Services University of Health Sciences, Bethesda, Maryland; 3 National Intrepid Center of Excellence, Walter Reed National Military Medical Center, Bethesda, Maryland; 4 Department of Preventive Medicine and Biostatistics, Uniformed Services University of Health Sciences, Bethesda, Maryland; 5 Department of Psychiatry, Yale University School of Medicine, New Haven, Connecticut; 6 Directorate of Behavioral Health, Walter Reed National Military Medical Center, Bethesda, Maryland; 7 USC Stevens Neuroimaging and Informatics Institute, Keck School of Medicine, University of Southern California, Los Angeles; 8 Department of Neurology, Walter Reed National Military Medical Center, Bethesda, Maryland

## Abstract

**Importance:**

Mild traumatic brain injury (mTBI) is the signature injury experienced by military service members and is associated with poor neuropsychiatric outcomes. Yet, there is a lack of reliable clinical tools for mTBI diagnosis and prognosis.

**Objective:**

To examine the white matter microstructure and neuropsychiatric outcomes of service members with a remote history of mTBI (ie, mTBI that occurred over 2 years ago) using diffusion tensor imaging (DTI) and neurite orientation dispersion and density imaging (NODDI).

**Design, Setting, and Participants:**

This case-control study examined 98 male service members enrolled in a study at the National Intrepid Center of Excellence. Eligible participants were active duty status or able to enroll in the Defense Enrollment Eligibility Reporting system, ages 18 to 60 years, and had a remote history of mTBI; controls were matched by age.

**Exposures:**

Remote history of mTBI.

**Main Outcomes and Measures:**

White matter microstructure was assessed using a region-of-interest approach of skeletonized diffusion images, including DTI (fractional anisotropy, mean diffusivity, radial diffusivity and axial diffusivity) and NODDI (orientation dispersion index [ODI], isotropic volume fraction, intra-cellular volume fraction). Neuropsychiatric outcomes associated with posttraumatic stress disorder (PTSD) and postconcussion syndrome were assessed.

**Results:**

A total of 65 male patients with a remote history of mTBI (mean [SD] age, 40.5 [5.0] years) and 33 age-matched male controls (mean [SD] age, 38.9 [5.6] years) were included in analysis. Compared with the control cohort, the 65 service members with mTBI presented with significantly more severe PTSD-like symptoms (mean [SD] PTSD CheckList-Civilian [PCL-C] version scores: control, 19.0 [3.8] vs mTBI, 41.2 [11.6]; *P* < .001). DTI and NODDI metrics were altered in the mTBI group compared with the control, including intra-cellular volume fraction of the right cortico-spinal tract (β = −0.029, Cohen *d* = 0.66; *P* < .001), ODI of the left posterior thalamic radiation (β = −0.006, Cohen *d* = 0.55; *P* < .001), and ODI of the left uncinate fasciculus (β = 0.013, Cohen *d* = 0.61; *P* < .001). In service members with mTBI, fractional anisotropy of the left uncinate fasciculus was associated with postconcussion syndrome (β = 5.4 × 10^−3^; *P* = .003), isotropic volume fraction of the genu of the corpus callosum with PCL-C (β = 4.3 × 10^−4^; *P* = .01), and ODI of the left fornix and stria terminalis with PCL-C avoidance scores (β = 1.2 × 10^−3^; *P* = .02).

**Conclusions and Relevance:**

In this case-control study of military-related mTBI, the results suggest that advanced magnetic resonance imaging techniques using NODDI can reveal white matter microstructural alterations associated with neuropsychiatric symptoms in the chronic phase of mTBI. Diffusion trends observed throughout widespread white matter regions-of-interest may reflect mechanisms of neurodegeneration as well as postinjury tissue scarring and reorganization.

## Key Points

**Question:**

Can the use of diffusion tensor imaging and neurite orientation dispersion and density imaging effectively detect white matter microstructural alterations in US military service members with a history of mild traumatic brain injury occurring more than 2 years ago, and can the results be used in monitoring neurobehavioral symptoms?

**Findings:**

In this case-control study of 98 military service members, both imaging tools detected widespread differences in white matter, with more sensitive results occurring with the use of neurite orientation dispersion and density imaging metrics through a region-of-interest approach. Notably, these white matter alterations were found to be associated with neurobehavioral symptoms.

**Meaning:**

These findings support the usefulness of advanced neuroimaging techniques in assessing microstructural changes related to military-related mild traumatic brain injury, and suggest that aberrant white matter properties can be used in monitoring progression or recovery during the chronic postinjury phase.

## Teaser

This case-control study of US military service members examines the sensitivity of 2 imaging techniques targeting white matter microstructure for diagnosis and prognosis of outcomes associated with mild traumatic brain injury (TBI).

## Introduction

Traumatic brain injury (TBI) is a significant health concern, particularly among military populations. According to the Defense and Veterans Brain Injury Center (DVBIC), more than 450 000 TBIs among US service members worldwide have been reported between 2000 and 2022, with over 80% of those TBIs being classified as mild (mTBI).^1^ Mounting evidence has demonstrated the neuropsychiatric consequences of mTBI, including chronic postconcussion symptoms and serious long-term effects on cognition, memory, mood, sleep, vision, and hearing.^2,3,4^ Furthermore, individuals with a history of mTBI are at higher risk for dementia, neurodegenerative diseases, psychiatric illness, and even mortality, indicating long-term progression of subclinical pathology that can manifest later in life.^5,6,7^

Despite mTBI lacking obvious clinical neuroimaging findings, making the detection of postinjury neurological changes challenging, there is increasing recognition that advanced neuroimaging techniques are promising biomarkers for its diagnosis, prognosis, and treatment monitoring.^8,9^ Specifically, diffusion-weighted imaging (DWI) utilizes the diffusion of water within brain tissue to infer microstructural tissue properties. One such advanced technique is diffusion tensor imaging (DTI), which provides a measure of the microstructural integrity of white matter fiber tracts through modeling of the DWI data sets. Within each voxel, DTI estimates specific diffusion variables, including mean diffusivity, fractional anisotropy, axial diffusivity, and radial diffusivity (Table 1).^29,30^

**Table 1.** Definition and Interpretation of Diffusion Tensor Imaging (DTI) and Neurite Orientation Dispersion and Density Imaging (NODDI) Metrics in the Context of Traumatic Brain Injury (TBI)

Metric	Description of measurement	Interpretation in TBI
**DTI diffusion metrics**		
Fractional anisotropy	Scalar value from 0 to 1, 0 = isotropic diffusion (equal in all directions)	Reduced fractional anisotropy,^10,11,12,13,14^ white matter tract disorganization^15^
Mean diffusivity	Scalar measure of water molecule diffusion within a voxel	Higher mean diffusivity, increased cellularity, edema, and necrosis^10,11,12,13,16^
Axial diffusivity	Magnitude of diffusion parallel to fiber tracts	Reduced axial diffusivity, axonal injury, diminished axonal caliber, or less coherent axon orientation^17^
Radial diffusivity	Coefficient of diffusion perpendicular to the main fiber orientation	Increased radial diffusivity,^18,19,20^ demyelination^21^
**NODDI diffusion metrics**		
ICVF	Fraction of tissue water restricted within neuritis, amount of neurites	Reduced ICVF, axonal loss or demyelination^22,23,24,25^; increased ICVF, axonal swelling in acute phase and regeneration in recovery phase^14,26,27^
ODI	Variability of neurite orientations, neurite spatial arrangement from 0 (parallel) to 1 (random)	Increased ODI, axonal dispersion or disorganization^23,25^; reduced ODI, reduced axonal complexity or glial scarring^14,28^
ISOVF	Fraction of tissue water confined within neurites	Increased ISOVF, tissue atrophy leading to expanded cerebrospinal fluid spaces^14,22^

Abbreviations: ICVF, intracellular volume fraction; ISOVF, isotropic volume fraction; ODI, orientation dispersion index.

Previous studies have demonstrated that white matter microstructural integrity, measured by DTI metrics, is perturbed following military-related mTBI.^31,32,33,34,35^ Additionally, DTI studies of mTBI have reported that microstructural white matter disruptions are associated with neurocognitive and behavioral deficits postinjury.^36,37,38^ However, studies of military mTBI have generally yielded varied findings on which white matter tracts are affected and whether fractional anisotropy is increased or decreased following injury.^39^ For instance, some studies report lower fractional anisotropy after remote mTBI,^40,41^ elevated fractional anisotropy,^42^ or a lack of significant mTBI effects on fractional anisotropy.^31,43,44,45,46^ These inconsistencies may be attributed to the variability in the mechanism (ie, different cellular alterations) and etiology of mTBI at different time points postinjury.

DTI metrics represent basic mathematical descriptions of diffusion that lack structural specificity and do not directly correspond to biophysically meaningful parameters of the underlying tissue. DTI assumes Gaussian diffusion within a single microstructural compartment and is thus insensitive to the complexity of white matter microstructure that is depicted through non-Gaussian models with multiple compartments.^47^ To address this limitation, a neurite orientation dispersion and density imaging (NODDI) model was created to offer more specific indices of tissue microstructure.^48^ NODDI leverages recent progress in high-performance magnetic field gradients for magnetic resonance imaging (MRI) scanners that can more specifically probe complex non-Gaussian properties of white matter diffusion (Table 1). A 2022 study^22^ on civilian subjects used NODDI to identify longitudinal white matter changes of declining neurite density after mTBI, suggesting axonal degeneration from diffuse axonal injury. The study concluded that NODDI metrics are more sensitive and specific biomarkers than DTI for white matter microstructural changes.

However, further investigation using both DTI and NODDI is needed in military populations to improve the health and well-being of service members at higher risk of TBIs and their associated consequences. To our knowledge, this case-control study is the first to leverage DTI and NODDI to examine subtle white matter neuropathological changes in military service members with mTBI as well as their association with neuropsychiatric symptomology in the chronic phase postinjury. We hypothesize that NODDI would be more sensitive and specific to microstructural changes than DTI and that diffusion metrics would be associated with neuropsychiatric symptoms. By combining advanced neuroimaging techniques and neuropsychiatric data, we aim to gain deeper insights into the underlying brain changes associated with military mTBI, ultimately enhancing our understanding and management of this complex condition.

## Methods

All participants were US military service members enrolled in the Walter Reed National Military Medical Center institutional review board–approved and HIPAA-compliant Neuroimaging Core project. All participants gave written informed consent. This cross-sectional study followed the Strengthening the Reporting of Observational Studies in Epidemiology (STROBE) reporting guideline.

### Participants and TBI Evaluation

Participants were scanned at the National Intrepid Center of Excellence at the Walter Reed National Military Medical Center. Inclusion criteria include active duty status or eligibility for the Defense Enrollment Eligibility Reporting system, age 18 to 60 years, male sex or female sex with no current pregnancy or breastfeeding. Exclusion criteria included patients with TBI unable to consent, actively enrolled in other treatment trials where this study would interfere, a history of prior severe neurologic or psychiatric conditions unrelated to the injury event or deployment (eg, meningioma, bipolar disorder), and patients with metal implants or shrapnel. Patients were targeted for recruitment and consent to the study if they had a history of mTBI (ie, mTBI group) or had no mTBI (ie, noninjured controls). Individuals were included in the mTBI group based on confirmed mTBI diagnosis with a history exceeding 2 years, male sex, and active duty status. Participants in the control group were matched for age, sex, and active duty status. Participants from both groups were excluded if they had incomplete PCL-C survey, DTI, or NODDI data. Diagnosis of TBI is described in eMethods in Supplement 1.

### Neuropsychiatric Assessments

The assessment of commonly affected neuropsychiatric domains after mTBI involved self-report surveys administered prior to the scan. These surveys included the PTSD Checklist–Civilian version (PCLC-C),^49^ Neurobehavioral Symptom Inventory (NSI),^50^ Generalized Anxiety Disorder-7 (GAD-7),^51^ and Patient Health Questionnaire-9 (PHQ-9).^52^ The PCL-C is a 17-item measure modeled after the *Diagnostic and Statistical Manual of Mental Disorders* (Fourth Edition, Text Revision) (*DSM-IV-TR*)^53^ symptom criteria for PTSD. Three cluster scores corresponding to the *DSM-IV-TR* symptom criteria were calculated: criterion B (reexperiencing cluster), criterion C (avoidance cluster), and criterion D (hyperarousal cluster). The NSI is a 22-item measure designed to evaluate self-reported postconcussion symptoms, such as headache, balance issues, and nausea, rated on a 5-point scale. A total score was obtained by summing the ratings for the 22 items, and 4 cluster scores were calculated as outlined by Vanderploeg and colleagues^54^: vestibular, somatosensory, cognitive, and affective clusters. The GAD-7 is a self-report survey consisting of 7 items designed to assess the severity of generalized anxiety disorder symptoms in individuals over the past 2 weeks. The PHQ-9 is a self-report questionnaire comprising 9 items used to measure the severity of depression symptoms and aid in diagnosing depressive disorders. While participants in both the mTBI and control groups completed the PCL-C, only patients with mTBI completed the NSI, GAD-7, and PHQ-9 surveys.

### MRI Acquisition and Image Processing

All subjects were scanned on a 3T MR750 scanner (General Electric) equipped with a 32-channel phased array head radiofrequency coil (MR Instruments). Whole-brain diffusion and structural MRI were obtained. Head motion was represented by framewise displacement measures of diffusion MRI (dMRI)^55^ and images were preprocessed with the protocol discussed in eMethods in Supplement 1.

DTI scalar images (ie, fractional anisotropy, mean diffusivity, axial diffusivity, and radial diffusivity) were created from dMRI with b-values between 0 and 1000 seconds/mm^2^ using the log-signal in 2 steps implemented in MRtrix3.^56^ Weighted least-squares with weights based on the empirical signal intensity followed by iterated weighted least-squares with weights determined by the signal predictions from the previous iteration using unconstrained optimization were applied to reconstructed DTI maps. NODDI parameters were calculated using the open-source tool AMICO,^57^ which yielded maps of intracellular volume fraction (ICVF), orientation dispersion index (ODI), and isotropic volume fraction (ISOVF) for each participant.^48^

### White Matter Region-of-Interest–Based Analysis

In a region-of-interest (ROI) analysis, the fractional anisotropy data were warped to the common FMRIB58 fractional anisotropy template in MNI152 standard space using the symmetric image normalization method implemented in the ANTs package^58^ using FMRIB Software Library’s toolbox tract-based spatial statistics (TBSS), a mean fractional anisotropy image was generated from all participant scans in this common space, creating a mean fractional anisotropy white matter skeleton representing common tracts across the entire group and thresholded at above 0.2 to exclude voxels containing gray matter and partial volume effects. The aligned fractional anisotropy volume was projected onto the skeleton by filling it with fractional anisotropy values from the nearest relevant tract center. Output images and the 0.2-thresholded skeleton were visually inspected for accuracy. The same nonlinear warping and skeleton projection steps were then applied to other whole brain mean diffusivity, radial diffusivity, axial diffusivity, ICVF, ODI, and ISOVF maps.

White matter main ROIs were examined by utilizing the Johns Hopkins University (JHU) ICBM-DTI-81 white matter Labeled Atlas^59^ in the standard MNI152 space. The JHU-ICBM-DTI-81 white matter labels atlas contains 46 white matter labels (eTable 1 in Supplement 1). To analyze these fasciculi, binary mask images corresponding to each tract were used to mask individual skeletonized maps that had been previously registered to the MNI (Montreal Neurological Institute) standard space. Regional values represented by the average voxel value within the selected JHU white matter tract masks were computed for each participant across all generated DTI and NODDI parameter maps.

### Statistical Analysis

All statistical analyses were performed using SPSS Statistics version 24.0 (IBM Corp) and R package 4.2.2 (R Project for Statistical Computing). Unpaired 2-sample *t* tests were used for demographic analysis between groups. χ^2^ tests compared the proportion of counts in each military-related category between groups with the expected proportions and the null hypothesis of equal proportions in each group. To compare DTI and NODDI parameters between mTBI and control groups, a generalized linear model (GLM) analysis was conducted with covariates of age and mean framewise displacement followed by nonparametric permutation test and randomization test (eMethods in Supplement 1). Variance inflation factor (VIF) was computed to assess the severity of multicollinearity in the OLS regression analysis.^60^ Finally, we performed a sensitivity analysis by comparing the variances between full and reduced OLS models and calculating the standardized regression coefficients (SRC), a global sensitivity indices based on linear or monotonic assumptions in the case of independent factors,^61,62^ and the Johnson indices, indices for correlated input relative importance by *R^2^* decomposition for linear regression models (eMethods in Supplement 1).^63,64^ The dichotomous receiver operating characteristic (ROC) curve analysis is described in eAppendix in Supplement 1. A 2-tailed *P* value at .05 was considered statistically significant.

## Results

### Demographic and Neuropsychiatric Assessment of Study Participants

A total of 98 study participants were included (all male; mean [SD] age, 40.0 [5.2] years); 33 participants had no history of mTBI (mean [SD] age, 39.1 [5.6] years) and 65 participants had a history of mTBI (mean [SD]age, 40.6 [5.0] years) (Table 2). Participants with a history of mTBI had significantly fewer education years compared with those in the control group (mean [SD] education, 14.7 [2.2] years vs 16.3 [2.8] years; *P* = .006). A significantly greater proportion of participants in the control group were in the Army compared with those in the mTBI group (22 of 33 [67%] vs 15 of 65 [23%]; *P* < .001). A significantly higher proportion of participants with mTBI were enlisted (53 of 65 [82%] vs 17 of 33 [52%]; *P* = .002). The mean (SD) mTBI count was 2.1 (1.4), with a mean (SD) time since the most recent injury being 14.2 (9.3) years. Most of the mechanisms causing mTBI were attributed to impacts (24 [37%]) and falls (20 [31%]). Additionally, 9 injuries (14%) resulted from blasts, 10 (15%) from motor vehicle accidents, 1 (2%) from gunshot wounds, and 1 (2%) from various other causes.

**Table 2.** Demographics, Military-Related Factors, Clinical Information, Neuropsychiatric Symptoms

Variable	Participants, No. (%)			*P* value
	Total (n = 98)	Control (n = 33)	mTBI (n = 65)	
Demographics				
Age, mean (SD), y	40.0 (5.2)	39.1 (5.6)	40.6 (5.0)	.23
Male sex	100 (100.0)	34 (100.0)	65 (100.0)	NA
Education, mean (SD), school years	15.2 (2.5)	16.3 (2.8)	14.7 (2.2)	.006
Military-related factors				
Service in Army branch	37 (37.8)	22 (66.7)	15 (23.1)	<.001
Enlisted military rank (enlisted 1-9)	70 (70.0)	17 (51.5)	53 (81.5)	.002
Duty status (active duty)	98 (100.0)	33 (100.0)	65 (100.0)	.16
Injury characteristics				
TBI count	NA	NA	2.1 (1.4)	NA
Time since most recent injury	NA	NA	14.1 (9.2)	NA
TBI mechanism				
Blow	NA	NA	24 (36.9)	NA
Fall	NA	NA	20 (30.8)	NA
Blast	NA	NA	9 (13.9)	NA
MVA	NA	NA	10 (15.4)	NA
Gunshot wound	NA	NA	1 (1.5)	NA
Other	NA	NA	1 (1.5)	NA
PTSD symptoms, mean (SD) PCL-C scores				
Total	33.50 (14.01)	19.00 (3.82)	40.86 (11.26)	<.001
B (reexperiencing)	8.08 (3.18)	5.40 (1.00)	9.45 (3.04)	<.001
C (avoidance)	12.87 (5.93)	7.45 (1.33)	15.62 (5.45)	<.001
D (hyperarousal)	12.55 (5.91)	6.15 (1.94)	15.80 (4.40)	<.001
Other neuropsychiatric symptoms				
NSI score, mean (SD)				
Total	NA	NA	36.1 (12.4)	NA
Somatosensory	NA	NA	8.7 (4.4)	NA
Affective	NA	NA	12.1 (4.6)	NA
Cognitive	NA	NA	9.1 (3.0)	NA
Vestibular	NA	NA	3.4 (2.0)	NA
PHQ-9 total	NA	NA	9.8 (4.8)	NA
GAD-7 total	NA	NA	11.6 (5.4)	NA

Abbreviations: GAD, Generalized Anxiety Disorder; mTBI, mild traumatic brain injury; MVA, motor vehicle accident; NA, not applicable; NSI, Neurobehavioral Symptom Inventory; PHQ, Patient Health Questionnaire; PCL-C, PTSD Checklist–Civilian Version; PTSD, posttraumatic stress disorder; TBI, traumatic brain injury.

Compared with the control group, participants with mTBI history had significantly higher PCL-C total scores (mean [SD] score, 40.9 [11.3] vs 19.0 [3.8]; *P* < .001). The mTBI group had a mean (SD) NSI total score of 36.1 (12.4), a somatosensory subscore of 8.4 (4.4), an affective subscore of 12.1 (4.6), a cognitive subscore of 9.1 (3.0), and a vestibular subscore of 3.4 (2.0). The majority of participants with mTBI history had moderately severe (20 participants [31%]) and severe (20 [31%]) anxiety as indicated by the GAD-7 scores as well as moderate (25 [39%]), moderately severe (6 [9%]), and severe (4 [6%]) depression as indicated by the PHQ-9 scores (eTable 2 in Supplement 1). Three service members with mTBI (4.6%) did not have NSI scores, which were replaced by imputation using R MICE. NSI, GAD-7, and PHQ-9 were not obtained in healthy controls.

### MRI Quality Control

There was no significant group difference of dMRI framewise displacement. This suggests participants with mTBI history had no greater head motion than controls during MRI examination.

### DTI and NODDI ROI-Based Comparisons and Their Association With Neuropsychiatric Symptoms

ROI analyses using GLMs with age as a covariate revealed widespread differences in DTI and NODDI metrics in various white matter regions (Table 3). Notably, more NODDI metrics were significantly different between control and mTBI groups compared with DTI metrics. Diffusion metrics of ROIs with the highest effect sizes between mTBI and control groups included ICVF of the right corticospinal tract (CST) (β = −0.029, *R^2^* = 0.136; *P* < .001), ODI of the left posterior thalamic radiation (PTR) (β = −6 × 10^−3^, *R^2^* = 0.253; *P* < .001) and ODI of the left uncinate fasciculus (UNC) (β = 0.013, *R^2^* = 0.125; *P* < .001).

**Table 3.** Results of Generalized Linear Models Assessing Mild Traumatic Brain Injury (mTBI) Effect on White Matter Region-of-Interest (ROIs) With Age as a Covariate^a^

Diffusion metric (ROI)^b^	Mean (SD), mm^2^/s		β (95% CI)	*t* value	*P* value	LRT	Cohen *d*	*R^2^*
	Control raw	mTBI raw						
Fractional anisotropy (left PTR)	0.6272 (0.0343)	0.6412 (0.0289)	0.016 (4 × 10^−3^ to 0.028)	2.56	.007	18.70	.45	0.190
Fractional anisotropy (left UNC)	0.6211 (0.0472)	0.5989 (0.0476)	−0.021 (−0.04 to −1.8 × 10^−3^)	−2.14	.02	13.48	.47	0.135
Mean diffusivity (fornix)	0.0012 (0.0003)	0.0011 (0.0002)	−1 × 10^−4^ (−2 × 10^−4^ to −4 × 10^−6^)	−2.04	.02	12.29	.35	0.106
Radial diffusivity (fornix)	0.0009 (0.0003)	0.0008 (0.0002)	−1 × 10^−4^ (−1.6 × 10^−4^ to −1.1 × 10^−4^)	−2.16	.02	13.66	.36	0.119
ICVF (right CST)	0.7442 (0.0480)	0.7171 (0.0371)	−0.029 (−0.046 to −0.012)	−3.35	<.001	30.15	.66	0.136
ICVF (left CST)	0.7433 (0.0428)	0.7249 (0.0404)	−0.021 (−0.038 to −0.004)	−2.45	.006	17.62	.45	0.126
ISOVF (genu CC)	0.1131 (0.0166)	0.1036 (0.0180)	−0.009 (−0.017 to −0.002)	−2.49	.007	17.81	.54	0.096
ISOVF (body CC)	0.0902 (0.0106)	0.0832 (0.0119)	−0.007 (−0.012 to −0.002)	−2.78	.002	21.79	.61	0.083
ISOVF (splenium CC)	0.0959 (0.0220)	0.0864 (0.0146)	−0.010 (−0.017 to −0.003)	−2.76	.003	21.43	.54	0.104
ISOVF (fornix)	0.3891 (0.1484)	0.3417 (0.1069)	−0.056 (−0.106 to −0.007)	−2.22	.01	14.38	.39	0.126
ISOVF (right CP)	0.1235 (0.0258)	0.1127 (0.0160)	−0.011 (−0.019 to −0.003)	−2.56	.005	18.75	.54	0.088
ISOVF (left SFO)	0.0444 (0.0153)	0.0522 (0.0186)	0.009 (0.001 to 0.016)	2.30	.009	15.34	.44	0.076
ODI (fornix)	0.1366 (0.1044)	0.1035 (0.0645)	−0.039 (−0.072 to −0.006)	−2.34	.01	15.90	.41	0.115
ODI (right CST)	0.1169 (0.0459)	0.0983 (0.0270)	−0.018 (−0.032 to −0.003)	−2.38	.01	16.42	.54	0.074
ODI (right ICP)	0.1422 (0.0262)	0.1333 (0.0196)	−0.010 (−0.019 to −0.0013)	−2.25	.01	14.78	.40	0.180
ODI (left SCP)	0.0830 (0.0196)	0.0756 (0.0107)	−0.007 (−0.013 to −0.0013)	−2.42	.007	16.92	.51	0.180
ODI (left PTR-L)	0.0673 (0.0118)	0.0621 (0.0076)	−0.006 (−0.009 to −0.002)	−3.17	<.001	27.41	.55	0.253
ODI (left fornix and stria terminalis)	0.1507 (0.0240)	0.1403 (0.0277)	−0.013 (−0.23 to −0.003)	−2.62	.004	19.52	.39	0.278
ODI (left UNC)	0.0705 (0.0156)	0.0837 (0.0240)	0.014 (0.005 to 0.023)	2.98	<.001	24.57	.61	0.125

Abbreviations: CC, corpus callosum; CP, cerebral peduncle; CST, corticospinal tract; ICP, inferior cerebellar peduncle; ISOVF, isotropic volume fraction; LRT, likelihood ratio test; ODI, orientation dispersion index; PTR, posterior thalamic radiation; SCP, superior cerebellar peduncle; SFO, superior fronto-occipital fasciculus; UNC, uncinate fasciculus.

^a^ ROI estimates determined by traumatic brain injury status and age.

^b^ Refer to eTable 1 in Supplement 1 for the definition of acronyms in the JHU-ICBM-DTI-81 white matter labels atlas. Expanded descriptions and interpretation of each measurement as they relate to TBI from the JHU-ICBM-DTI-81 available in eTable 1 in Supplement 1.

When assessing the association with neuropsychiatric symptoms, NSI cognitive subscores were associated with fractional anisotropy of the left UNC (β = 5.4 × 10^−3^; *P* = .003); PCL-C total scores were associated with ISOVF of the genu of corpus callosum (β = 4.3 × 10^−4^; *P* = .01); PCL-C C avoidance subscores were associated with ODI of the left fornix (crus) and stria terminalis (β = 1.2 × 10^−3^; *P* = .02). VIF, an index measuring how much the variance of an estimated regression coefficient is increased because of collinearity, of all models were less than 1.2 (Table 4). Sensitivity analysis of the 3 regression models revealed that the full model (3 independent variables) explained the variances of diffusion metrics better than those of the reduced model (2 independent variables) with an SRC of 0.335, 0.266, and 1.000, and *R^2^* of 0.091, 0.067, and 0.867 (Johnson indices) for NSI cognitive, PCL-C total scores and PCL-C C avoidance subscores, respectively (eTable 4 in Supplement 1). ROC curve analysis is available in eResults in Supplement 1.

**Table 4.** Summary of Significant Diffusion Metrics Regions-of-Interest (ROIs) Associated With Neuropsychiatric Symptoms (Traumatic Brain Injury Only)

Model	β (SE)	*t* value	*P* value	LRT	VIF	*R^2 ^* ^a^
**Fractional anisotropy of left UNC = ** **NSI cognitive + age + mean displacement**						
NSI cognitive	5.4 × 10^−3^ (1.9 × 10^−3^)	2.77	.003	57.9	1.06	0.195
Age	9.2 × 10^−4^ (1.1 × 10^−3^)	0.79	.21	36.9	1.08	
Mean displacement	−0.130 (0.046)	−2.82	.003	103.5	1.10	
**ISOVF of genu CC = PCLC total + age + mean displacement**						
PCLC total	4.3 × 10^−4^ (1.9 × 10^−4^)	2.15	.01	48.3	1.01	0.079
Age	1.8 × 10^−4^ (4.6 × 10^−4^)	−0.40	.35	11.4	1.06	
Mean displacement	−0.014 (0.018)	−0.776	.21	17.4	1.07	
**ODI of left fornix/ST = PCLC-C + age + mean displacement**						
PCLC-C	1.2 × 10^−3^ (5.5 × 10^−4^)	2.09	.02	33.6	1.02	0.289
Age	1.9 × 10^−3^ (6.2 × 10^−4^)	3.06	.001	162.2	1.06	
Mean displacement	0.55 (0.024)	2.26	.01	83.9	1.07	

Abbreviations: CC, corpus callosum; ISOVF, isotropic volume fraction; LRT, likelihood ratio test; NSI, Neurobehavioral Symptom Inventory; PCLC, Posttraumatic Stress Disorder Checklist–Civilian version; ST, stria terminalis; UNC, uncinate fasciculus; VIF, variance inflation factor.

^a^ *R^2^* calculated by 1 – (SS residual / SS total), where SS is the sum of the square.

## Discussion

This case-control study investigated diffusion parameters of white matter in military service members with and without remote mTBI. To the best of our knowledge, this is the first study to report white matter microstructural changes indicated by DTI and NODDI metrics using an ROI-based approach in the chronic phase of mTBI among military service members. Our study supports previous evidence that mTBI can have long-term effects on white matter microstructure and neuropsychiatric symptoms related to PTSD, postconcussion syndrome, anxiety, and depression.^65,66,67,68^

The ROI analysis yielded extensive white matter alterations in the mTBI group that were also linked to neuropsychiatric symptoms. Notably, the analysis revealed DTI and NODDI trends of increased anisotropic and parallel diffusion, implicating mechanisms of inflammation, glial cell activation, and tissue scarring or reorganization.^69,70^ For example, there was increased fractional anisotropy and axial diffusivity, as well as decreased ODI in the left PTR. It is often assumed that lower fractional anisotropy values may correspond to a reduction of the white matter microstructural integrity.^71^ However, emerging evidence suggests that higher fractional anisotropy and axial diffusivity values can be attributed to factors such as cytotoxic edema during the acute phase following injury^72,73,74^ as well as glial scarring and a manifestation of recovery and/or compensation in the chronic phase,^69,72,74,75,76^ or simply in the voxels with less fiber crossings. In these conditions, the interpretation of higher fractional anisotropy values as solely indicative of increased white matter integrity may not hold true. Some studies indicated that astrocytes undergoing structural remodeling postinjury, thereby driving glial scarring, lead to anisotropic tissue microstructure causing an increase in fractional anisotropy.^69,70,77^ Notably, a 2011 study^69^ performing a histological analysis showed that glial fibrillary acidic protein (GFAP), which is a marker for gliosis, was significantly positively correlated with fractional anisotropy and axial diffusivity. Furthermore, decreased mean diffusivity, radial diffusivity, ISOVF, and ODI were observed in the fornix, with ODI values being associated with PCL-C scores. Reduced ISOVF in the corpus callosum was also associated with PCL-C total. Altogether, our findings of compromised white matter in these key tracts complement previous findings that also showed white matter changes in military service members with a remote history of mTBI.^41,78^

Alternatively, our study also revealed diffusion trends implicating mechanisms of neurodegeneration. For example, decreased ICVF in right and left CST suggests reduced neurite density; reduced fractional anisotropy with increased ODI in the left UNC may suggest axonal degeneration with compensatory neural sprouting.^79,80,81^ These findings are consistent with our 2023 study^82^ investigating fiber-specific structural changes in a larger cohort of military service members after a remote brain injury. Importantly, fractional anisotropy was significantly reduced in the left UNC and was associated with cognitive-related PCS scores. This anatomically aligns with the significance of UNC in key neural circuitry involving the entorhinal and amygdala, which plays a pivotal role in memory formation and emotion regulation.^83,84^ Postconcussion symptoms, PTSD symptoms, and neuropsychological function have been shown to be associated with compromised fronto-limbic neurocircuitry in chronic mTBI.^85,86^

While statistical significance shows that an association exists in a study, effect size indicates the practical significance of a research outcome. *R^2^* determines the proportion of variance in the dependent variable that can be explained by the independent variable, representing the goodness of fit. Our results show that the ICVF of the right CST had a high Cohen *d* for group difference but a low *R^2^* value, while the ODI of left fornix and stria terminalis had a high SRC and *R^2^* but a relatively low Cohen *d*. These findings might help explain why diffusion metrics exhibit low dichotomous discrimination.

Lastly, ROC curve analyses indicated that self-reported neuropsychiatric symptoms were more effective in distinguishing between the mTBI and control groups compared with imaging metrics, which did not provide significant discriminatory power. Particularly, PTSD symptoms were successful in classifying participants with mTBI history from controls in this patient population. Prior studies have highlighted the increased risk of comorbid PTSD and mTBI among military service members compared with civilians.^87,88,89,90^ This emphasizes the importance of assessing PTSD symptoms and associated neuropsychiatric symptoms in the management of military-related mTBI. Indeed, while neuroimaging metrics did not show superior discriminatory ability, they did facilitate the identification of potential lesions associated with neuropsychiatric outcomes, which can be used to predict clinical progression and determine the most suitable treatment approach.^91,92^

### Limitations

This study had several limitations. This was a cross-sectional case-control study with a relatively limited sample size, which offers only a snapshot of the neural factors associated with neuropsychiatric symptoms in the chronic phase postinjury. Although the sample size was similar to other studies using NODDI to examine mTBI,^22,23,28,93,94^ further analysis with a larger sample is necessary to validate the findings. Additionally, there were missing clinical data that might have influenced the results, including past medical history, preexisting conditions, medications, treatments, or interventions received. Finally, a causal relationship between white matter microstructural alterations and neuropsychiatric symptom presentation cannot be assumed. Thus, these findings should be considered as suggestive of a relationship that should be replicated in other studies.

## Conclusions

In this case-control study of military-related mTBI, our results showed that DTI and NODDI can detect microstructural white matter alterations in the chronic phase of mTBI, implicating inflammatory and neurodegenerative processes. Moreover, our findings suggest that NODDI might offer greater sensitivity than DTI in identifying alterations during the chronic phase. Consequently, combining NODDI with DTI parameters in future research could yield valuable insights. Specifically, our results suggest that mTBI in military service members is characterized by widespread differences in diffusion parameters of white matter tracts important for cognitive and emotional processing, including the corpus callosum, CST, UNC, and fornix. Our findings indicate that DTI and NODDI metrics can provide valuable pathophysiological insights into the long-term neuropsychiatric consequences of mTBI.

## Notes

Supplement 1.
eMethods.
**eAppendix.** ROC Curve Analysis **eFigure.** ROC Curves of PCL-C Scores and NODDI/DTI Metrics Classifying mTBI From Control **eTable 1.** Index of ROIs From the JHU-ICBM-DTI-81 WM Labels Atlas **eTable 2.** Severity Ratings of GAD-7 and PHQ-9 **eTable 3.** Results of Sensitivity Analysis of Regression Models (TBI Only, n = 65) **eTable 4.** Diagnostic Sensitivity and Specificity of PCL-C Scores and Diffusion Parameters for Classifying mTBI From Control
eReferences.

Supplement 2.
Data Sharing Statement
