# Error in Byline and Author Contributions

**DOI:** 10.1001/jamanetworkopen.2024.20754

**Published:** 2024-06-07

In the Original Investigation titled “White Matter Alterations in Military Service Members With Remote Mild Traumatic Brain Injury,”^1^ published April 18, 2024, a coauthor was not included in the submitted manuscript. Dr Hosung Kim and his authorship contributions have been added. This article has been corrected.^1^
