# Supplementary material for: White Matter Alterations in Military Service Members With Remote Mild Traumatic Brain Injury
Source: JAMA Netw Open. 2024 Apr 18;7(4):e248121. doi: 10.1001/jamanetworkopen.2024.8121 (PMC11161843; doi:10.1001/jamanetworkopen.2024.8121)
Supplement: Supplement 1. — eMethods. eAppendix. ROC Curve Analysis eFigure. ROC Curves of PCL-C Scores and NODDI/DTI Metrics Classifying mTBI From Control eTable 1. Index of ROIs From the JHU-ICBM-DTI-81 WM Labels Atlas eTable 2. Severity Ratings of GAD-7 and PHQ-9 eTable 3. Results of Sensitivity Analysis of Regression Models (TBI Only, n = 65) eTable 4. Diagnostic Sensitivity and Specificity of PCL-C Scores and Diffusion Parameters for Classifying mTBI From Control eReferences. [file jamanetwopen-e248121-s001.pdf]

Supplemental Online Content

Kim S, Ollinger J, Song C, et al. White matter alterations in military service members with remote mild traumatic brain injury. *JAMA Netw Open.* 2024;7(4):e248121. doi:10.1001/jamanetworkopen.2024.8121

eMethods.

eAppendix. ROC Curve Analysis

eFigure. ROC Curves of PCL-C Scores and NODDI/DTI Metrics Classifying mTBI From Control

eTable 1. Index of ROIs From the JHU-ICBM-DTI-81 WM Labels Atlas

eTable 2. Severity Ratings of GAD-7 and PHQ-9

eTable 3. Results of Sensitivity Analysis of Regression Models (TBI Only, n = 65)

eTable 4. Diagnostic Sensitivity and Specificity of PCL-C Scores and Diffusion Parameters for Classifying mTBI From Control

eReferences.

This supplemental material has been provided by the authors to give readers additional information about their work.

© 2024 Kim S et al. *JAMA Network Open.*

eMethods.

Diagnosis of TBI

Diagnosis of TBI was based on information gathered via medical record review and a comprehensive lifetime TBI history interview completed by a Masters- or PhD-level clinical research personnel who were trained to evaluate the presence and severity of TBI. The TBI history interview consisted of the Ohio State University TBI identification method.[1] Final determination and classification of TBI severity was undertaken by consensus, giving consideration to all information, during case conferencing with the interviewer and a clinician/scientist trained in neuropsychology and TBI.

MRI acquisition.

Whole-brain diffusion MRI was performed using combined Simultaneous Multislice (SMS, factor=3) and in-plane Parallel (acceleration factor=2) single-shot echo planar pulse sequence using 270 diffusion-encoding directions, acquired at b = 1000, 2000, and 3000 s/mm^2, and eighteen acquisitions at b = 0 s/mm^2 with 1.7-mm isotropic voxels. A sparse and optimal acquisition design[2] was applied to optimize the diffusion measurements in each shell and maximum uniformity of diffusion measurement directions for diffusion MRI multiple-shell acquisition. Two dMRI sets were acquired using blip-up and down phase-encoding acquisition approach. Sagittal three-dimensional (3D) inversion recovery fast spoiled gradient recalled echo T1-weighted were acquired with BRAVO sequence. T2*-weighted images were acquired using sagittal 3D gradient echo sequence.

Preprocessing of dMRI data included dMRI de-noising, Gibbs ringing removal and correction of motion and eddy current artifacts using software from the TORTOISE toolkits.[3] Susceptibility-induced off-resonance fields were estimated using the different phase-encoded images, and an undistorted structural T2-weighted MRI image was aligned to the AC-PC plane

© 2024 Kim S et al. *JAMA Network Open.*

using the HCP Preprocessing Pipeline (https://github.com/Washington-University/HCPpipelines) and used to improve registration accuracy. EPI geometric distortion correction was performed using pairs of diffusion data sets acquired with opposite phase encoding (blip-up and blip-down acquisitions).[4] dMRI images were finally upsampled to an isotropic voxel size of 1.0 mm^3, and two sets of dMRI were averaged. The b-matrices were rotated accordingly. Framewise Displacement (FD) was measured using the implementation in Nipype (https://nipype.readthedocs.io/en/latest/), following the definitions by, wherein all position measures were differentiated in dMRI volume frames by backwards differences, rotational measures were converted to arc displacement at 5 cm radius, and the sum of the absolute value of these measures was calculated.[5]

Statistical Analyses

Permutation inference Generalized Linear Model

Specifically, GLM generalized the ordinary least squares (OLS) by allowing Chi-Square distribution of the residuals, and optimized the likelihood function by allowing a t-test based on an estimated error of the coefficients. Permutation inference GLMs applied OLS with covariates of age and FD to examine the relationship between the DTI/NODDI metrics and neuropsychiatric symptoms.

To assess goodness-of-fit, likelihood ratio test (LRT) statistic, comparing the log likelihoods between the null and the full models, and R-squared static, representing the percentage of variation of diffusion metrics explained by the independent variables, were calculated.

The nonparametric statistical test was performed in the following way[6]:

(1) Collect the trials of the two experimental conditions in a single set.

© 2024 Kim S et al. *JAMA Network Open.*

(2) Randomly draw as many trials from this combined data set as there were trials in condition 1 and place those trials into subset 1. Place the remaining trials in subset 2. The result of this procedure was called a random partition.

(3) Calculate the test statistic on this random partition.

(4) Repeat steps 2 and 3 a large number of times and constructed a histogram of the test statistics.

(5) From the test statistic that was actually observed and the histogram in step 4, calculated the proportion of random partitions that resulted in a larger test statistic than the observed one. This proportion was called the *p*-value.

(6) If the *p*-value was smaller than the critical alpha-level, e.g. 0.05, then concluded that the data in the two experimental conditions were significantly different.

Calculate the Monte Carlo p-values

The Monte Carlo estimate, the Monte Carlo *p*-values, were obtained by repeating steps 2 and 3 above at 5000 times followed by comparing these random test statistics (i.e., draws from the permutation distribution) with the observed test statistic. The Monte Carlo estimate of the permutation *p*-value is the proportion of random partitions in which the observed test statistic was larger than the value drawn from the permutation distribution. Because the Monte Carlo *p*-value has a binomial distribution, its accuracy can be quantified by means of the confidence interval for a binomial proportion.[7]

Sensitivity analysis

Residual sum of squares *and* R-squared, as well as the F values (the ratio of variances of the full and the reduced OLS models) were computed and compared between the full and the reduced OLS models. We computes the Standardized Regression Coefficients (SRC) which are the normalized linear regression coefficients, and Johnson indices which allocate a share of R^2

© 2024 Kim S et al. *JAMA Network Open.*

to each input based on the relative weight allocation (RWA) system, in the case of dependent or correlated inputs.

Receiver operating characteristic (ROC) curve analysis

Dichotomus ROC curve analysis was employed to compare diffusion metrics and differentiate mTBI patients from controls based on the WM microstructural differences identified by DTI/NODDI metrics. The area under each ROC curve (AUC) was calculated to determine the optimal discriminative performance among DTI/NODDI diffusion metrics, along with PCL-C scores. The threshold value for a significant diffusion metric to distinguish mTBI patients from healthy controls was identified by maximizing the Youden index.[8]

© 2024 Kim S et al. *JAMA Network Open.*

eAppendix. ROC Curve Analysis

In the ROC curve analysis, the area under the curve (AUC) was calculated to evaluate the performance of different variables in distinguishing between mTBI and control subjects. ROC curves were generated from PCL-C total and sub-scores, as well as the significantly different DTI/NODDI ROI and TBSS clusters. PCL-C total score had the highest AUC of 0.981 ($p<.001$), followed by PCL-C D hyperarousal sub-score (AUC=0.980, $p<.001$), PCL-C C avoidance sub-score (AUC=0.960, $p<.001$), and PCL-C B re-experiencing sub-score (AUC=0.905, $p<.001$) **(eFigure 1 and eTable 4)**. For PCL-C total score, the optimal cutoff value was 23.0 with a sensitivity of 96.9% and a specificity of 87.9%.

© 2024 Kim S et al. *JAMA Network Open.*

eFigure. ROC curves of PCL-C Scores and NODDI/DTI metrics classifying mTBI from control

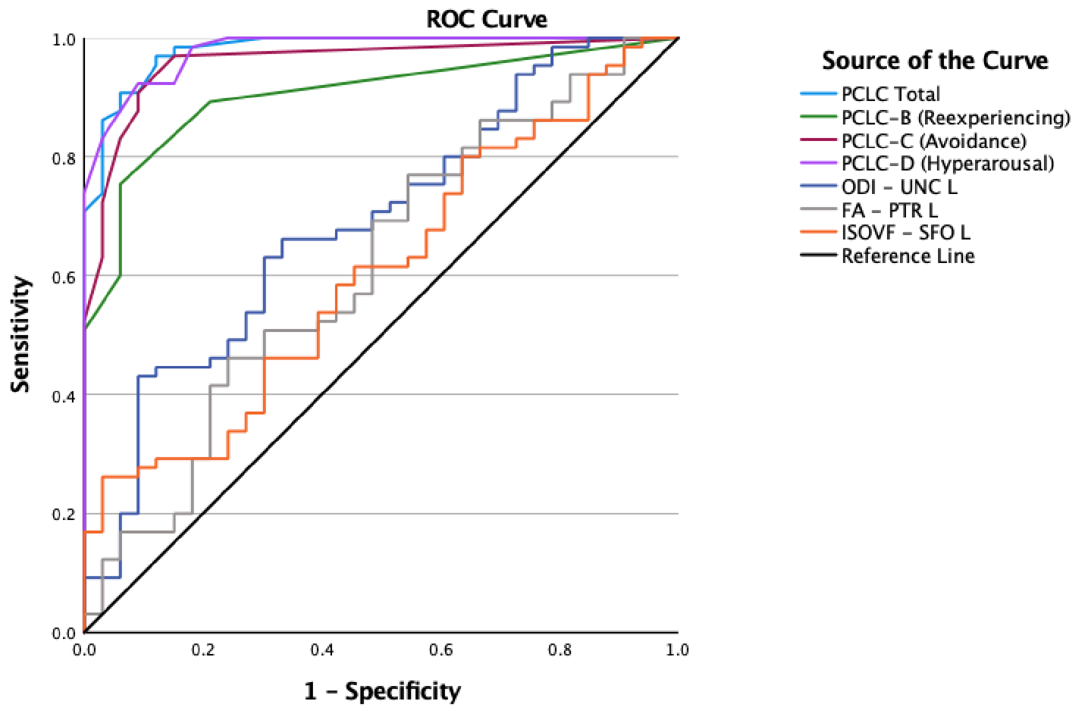

eFigure. ROC curves of PCL-C Scores and NODDI/DTI metrics classifying mTBI from control. PCL-C total and sub scores as well as neuroimaging metrics with the highest AUC values were included in the graph. PCL-C total score had the highest AUC of 0.981 (p<.001); the optimal cutoff value was 23.0 with a sensitivity of 96.9% and a specificity of 87.9%. Among all imaging metrics, ODI of the ROI UNC-L had the highest AUC of 0.698 (p=.001); the optimal cutoff value was 0.0852 with a sensitivity of 43.0% and specificity of 90.3%.

© 2024 Kim S et al. *JAMA Network Open.*

eTable 1. Index of ROIs From the JHU-ICBM-DTI-81 WM Labels Atlas

1. GCC - Genu of Corpus Callosum<br>2. BCC - Body of Corpus Callosum<br>3. SCC - Splenium of Corpus Callosum<br>4. FX - Fornix<br>5. CST-R - Corticospinal Tract Right<br>6. CST-L - Corticospinal Tract Left<br>7. ML-R - Middle Cerebellar Peduncle Right<br>8. ML-L - Middle Cerebellar Peduncle Left<br>9. ICP-R - Inferior Cerebellar Peduncle Right<br>10. ICP-L - Inferior Cerebellar Peduncle Left<br>11. SCP-R - Superior Cerebellar Peduncle Right<br>12. SCP-L - Superior Cerebellar Peduncle Left<br>13. CP-R - Cerebral Peduncle Right<br>14. CP-L - Cerebral Peduncle Left<br>15. ALIC-R - Anterior Limb of Internal Capsule Right<br>16. ALIC-L - Anterior Limb of Internal Capsule Left<br>17. PLIC-R - Posterior Limb of Internal Capsule Right<br>18. PLIC-L - Posterior Limb of Internal Capsule Left<br>19. RLIC-R - Retrolenticular Part of Internal Capsule Right<br>20. RLIC-L - Retrolenticular Part of Internal Capsule Left<br>21. ACR-R - Anterior Corona Radiata Right<br>22. ACR-L - Anterior Corona Radiata Left	23. SCR-R - Superior Corona Radiata Right<br>24. SCR-L - Superior Corona Radiata Left<br>25. PCR-R - Posterior Corona Radiata Right<br>26. PCR-L - Posterior Corona Radiata Left<br>27. PTR-R - Posterior Thalamic Radiation Right<br>28. PTR-L - Posterior Thalamic Radiation Left<br>29. SS-R - Sagittal Stratum Right<br>30. SS-L - Sagittal Stratum Left<br>31. EC-R - External Capsule Right<br>32. EC-L - External Capsule Left<br>33. CGC-R - Cingulum Cingulate Gyrus Right<br>34. CGC-L - Cingulum Cingulate Gyrus Left<br>35. CGH-R - Cingulum Hippocampus Right<br>36. CGH-L - Cingulum Hippocampus Left<br>37. FX/ST-R - Fornix (crus)/Stria terminalis Right<br>38. FX/ST-L - Fornix (crus)/Stria terminalis Left<br>39. SLF-R - Superior Longitudinal Fasciculus Right<br>40. SLF-L - Superior Longitudinal Fasciculus Left<br>41. SFO-R - Superior fronto-occipital fasciculus Right<br>42. SFO-L - Superior fronto-occipital fasciculus Left<br>43. IFO-R - Inferior Fronto-Occipital Fasciculus Right<br>44. IFO-L - Inferior Fronto-Occipital Fasciculus Left<br>45. UNC-R - Uncinate Fasciculus Right<br>46. UNC-L - Uncinate Fasciculus Left

© 2024 Kim S et al. *JAMA Network Open.*

eTable 2. Severity Ratings of GAD-7 and PHQ-9

GAD-7 Rating	Frequency	Percent
**Severe**	20	30.8
**Moderately Severe**	20	30.8
**Moderate**	18	27.7
**Mild**	5	7.7
**None**	2	3.1
**Total**	65	100.0
**PHQ-9 Rating**	**Frequency**	**Percent**
**Severe**	4	6.3
**Moderately Severe**	6	9.4
**Moderate**	25	39.1
**Mild**	21	32.8
**None**	1	1.6
**Total**	64	100.0

© 2024 Kim S et al. *JAMA Network Open.*

eTable 3. Results of Sensitivity Analysis of Regression Models (TBI only, n=65)

**Full model: L FA UNC ~ NSI Cognitive + Age + Mean Displacement**<br>**Reduced model: L FA UNC ~ Age + Mean Displacement**				
Model	***SRC***	***Johnson Indices***	***F-Value ANOVA (Full model, Reduced model)***	***P-Value ANOVA (Full model, Reduced model)***
NSI Cognitive	0.335	0.091	7.96	0.006
Age	0.095	0.003		
Mean displacement	-0.332	0.085		
**Full model: ISOVF GCC ~ PCLC Total + Age + Mean Displacement**<br>**Reduced model: ISOVF GCC ~ Age + Mean Displacement**				
Model	***SRC***	***Johnson Indices***	***F-Value ANOVA (Full model, Reduced model)***	***P-Value ANOVA (Full model, Reduced model)***
PCLC Total	0.266	0.067	4.63	*0.03*
Age	-0.051	0.004		
Mean displacement	-0.098	0.008		
**Full model: L ODI FX ST ~ PCLC-C + Age + Mean Displacement**<br>**Reduced model: L ODI FX ST ~ Age + Mean Displacement**				
Model	***SRC***	***Johnson Indices***	***F-Value ANOVA (Full model, Reduced model)***	***P-Value ANOVA (Full model, Reduced model)***
PCLC-C	1.000	0.867	4.35	*0.04*
Age	6.85e-17	0.074		
Mean displacement	2.40e-16	0.059		

© 2024 Kim S et al. *JAMA Network Open.*

Table 4. Diagnostic Sensitivity and Specificity of PCL-C Scores and Diffusion Parameters for Classifying mTBI From Control

Test Result Variable(s)	AUC (95% CI)	SE	P-value	Max YI	Cutoff value	Sensitivity	Specificity
**PCL-C Total**	0.980 (0.961 to 1.000)	0.011	<.001	0.848	23.0	0.97	0.88
**PCL-C D (Hyperarousal)**	0.979 (0.962 to 1.000)	0.011	<.001	0.693	6.50	0.75	0.94
**PCL-C C (Avoidance)**	0.959 (0.920 to 0.998)	0.020	<.001	0.818	7.50	0.97	0.85
**PCL-C B (Reexperiencing)**	0.904 (0.843 to 0.964)	0.031	<.001	0.832	9.50	0.92	0.91
**ODI: UNC-L**	0.689 (0.579 to 0.799)	0.056	.002	0.340	0.0852	0.43	0.91
**FA: PTR-L**	0.615 (0.496 to 0.735)	0.061	.06	0.224	0.6210	0.77	0.45
**ISOVF: SFO-L**	0.605 (0.490 to 0.720)	0.059	.09	0.231	0.0652	0.26	0.97
**MD: FX**	0.435 (0.304 to 0.566)	0.067	.3	0.151	0.0009	0.97	0.18
**RD: FX**	0.429 (0.297 to 0.561)	0.068	.3	0.151	0.0006	0.94	0.21
**ODI: ICP-R**	0.407 (0.284 to 0.531)	0.063	.1	0.028	0.1113	0.88	0.15
**ISOVF: FX**	0.394 (0.267 to 0.521)	0.065	.09	0.090	0.1882	0.97	0.12
**ODI: FX**	0.388 (0.260 to 0.516)	0.065	.07	0.075	0.0627	0.92	0.15
**ISOVF: CP-R**	0.386 (0.264 to 0.509)	0.062	.07	0.000	0.0000	1.00	0.00
**ODI: SCP-L**	0.385 (0.263 to 0.507)	0.062	.06	0.015	0.0564	0.98	0.03
**ODI: CST-R**	0.383 (0.264 to 0.502)	0.061	.06	0.015	0.0625	0.98	0.03
**ICSVF: CST-L**	0.382 (0.261 to 0.502)	0.062	.06	0.015	0.8284	0.02	1.00
**FA: UNC-L**	0.380 (0.262 to 0.499)	0.060	.05	0.000	0.0000	1.00	0.00
**ODI: PTR-L**	0.370 (0.246 to 0.494)	0.063	.04	0.029	0.0516	0.94	0.09
**ODI: FX/ST-L**	0.369 (0.256 to 0.483)	0.058	.04	0.046	0.2058	0.05	1.00
**ICVF: CST-R**	0.348 (0.232 to 0.464)	0.059	.01	0.000	0.0000	1.00	0.00
**ISOVF: GCC**	0.333 (0.225 to 0.441)	0.055	.007	0.015	0.1736	0.02	1.00
**ISOVF: SCC**	0.332 (0.208 to 0.457)	0.064	.007	0.075	0.0690	0.92	0.15
**ISOVF: BCC**	0.317 (0.206 to 0.428)	0.056	.003	0.047	0.1050	0.08	0.97

*Refer to eTable 1 in Supplementary for the definition of acronyms in the JHU-ICBM-DTI-81 WM labels atlas, an expanded description of each measurement and interpretation of each measurement as they relate to TBI.

Abbreviations: AUC = Area under the curve, CI = Confidence Interval, mTBI = mild traumatic brain injury, PCL-C = PTSD Checklist – Civilian Version, SE = Standard Error, YI = Youden Index.

© 2024 Kim S et al. *JAMA Network Open.*

eReferences.

1. Corrigan JD, Bogner J. Initial reliability and validity of the Ohio State University TBI identification method. *The Journal of head trauma rehabilitation*. 2007;22(6):318-329.
2. Koay CG, Özarslan E, Johnson KM, Meyerand ME. Sparse and optimal acquisition design for diffusion MRI and beyond. *Medical physics*. 2012;39(5):2499-2511.
3. Pierpaoli C, Walker L, Irfanoglu MO, et al. TORTOISE: an integrated software package for processing of diffusion MRI data. Stockholm; 2010:
4. Irfanoglu MO, Modi P, Nayak A, Hutchinson EB, Sarlls J, Pierpaoli C. DR-BUDDI (Diffeomorphic Registration for Blip-Up blip-Down Diffusion Imaging) method for correcting echo planar imaging distortions. *Neuroimage*. 2015;106:284-299.
5. Power JD, Mitra A, Laumann TO, Snyder AZ, Schlaggar BL, Petersen SE. Methods to detect, characterize, and remove motion artifact in resting state fMRI. *Neuroimage*. 2014;84:320-341.
6. Maris E, Oostenveld R. Nonparametric statistical testing of EEG-and MEG-data. *Journal of neuroscience methods*. 2007;164(1):177-190.
7. Ernst MD. Permutation methods: a basis for exact inference. *Statistical Science*. 2004:676-685.
8. Fluss R, Faraggi D, Reiser B. Estimation of the Youden Index and its associated cutoff point. *Biometrical Journal: Journal of Mathematical Methods in Biosciences*. 2005;47(4):458-472.

© 2024 Kim S et al. *JAMA Network Open.*
