# Supplementary material for: White Matter Alterations in Military Service Members With Remote Mild Traumatic Brain Injury
Source: JAMA Netw Open. 2024 Apr 18;7(4):e248121. doi: 10.1001/jamanetworkopen.2024.8121 (PMC11161843; doi:10.1001/jamanetworkopen.2024.8121)
Supplement: Supplement 2. — Data Sharing Statement [file jamanetwopen-e248121-s002.pdf]

Data Sharing Statement

Kim. White Matter Alterations in Military Service Members With Remote Mild Traumatic Brain Injury. *JAMA Netw Open*. Published April 18, 2024. doi:10.1001/jamanetworkopen.2024.8121

Data
Data available: Yes
Data types: Deidentified participant data
How to access data: Email: pinghongyeh@gmail.com
When available: With publication

Supporting Documents
Document types: Statistical/analytic code, Informed consent form
How to access documents: pinghongyeh@gmail.com
When available: With publication

Additional Information
Who can access the data: anyone requesting the data
Types of analyses: for non-commercial purpose
Mechanisms of data availability: with investigator support and with a signed data access agreement
